# Tubulin-Targeted Therapy in Melanoma Increases the Cell Migration Potential by Activation of the Actomyosin Cytoskeleton—An In Vitro Study

**DOI:** 10.1021/acsbiomaterials.4c01226

**Published:** 2024-10-22

**Authors:** Marcin Luty, Renata Szydlak, Joanna Pabijan, Joanna Zemła, Ingrid H. Oevreeide, Victorien E. Prot, Bjørn T. Stokke, Malgorzata Lekka, Bartlomiej Zapotoczny

**Affiliations:** † Institute of Nuclear Physics, Polish Academy of Sciences, Krakow PL-31342, Poland; ‡ Biophysics and Medical Technology, Department of Physics, NTNU The Norwegian University of Science and Technology, Trondheim NO-7491, Norway; § Biomechanics, Department of Structural Engineering, NTNU The Norwegian University of Science and Technology, Trondheim NO-7491, Norway

**Keywords:** melanoma, invasiveness, colchicine, cytoskeleton, actin, tubulin

## Abstract

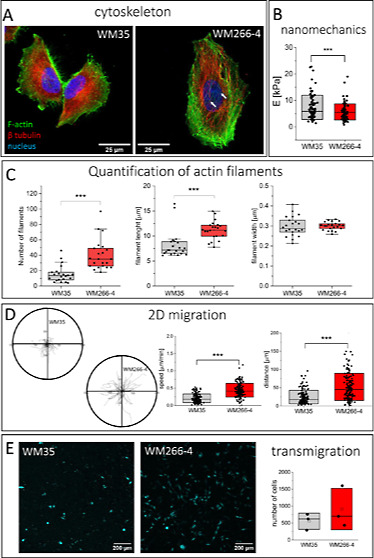

One of the most dangerous aspects of cancers is their ability to metastasize, which is the leading cause of death. Hence, it holds significance to develop therapies targeting the eradication of cancer cells in parallel, inhibiting metastases in cells surviving the applied therapy. Here, we focused on two melanoma cell lines—WM35 and WM266-4—representing the less and more invasive melanomas. We investigated the mechanisms of cellular processes regulating the activation of actomyosin as an effect of colchicine treatment. Additionally, we investigated the biophysical aspects of supplement therapy using Rho-associated protein kinase (ROCK) inhibitor (Y-27632) and myosin II inhibitor ((−)-blebbistatin), focusing on the microtubules and actin filaments. We analyzed their effect on the proliferation, migration, and invasiveness of melanoma cells, supported by studies on cytoskeletal architecture using confocal fluorescence microscopy and nanomechanics using atomic force microscopy (AFM) and microconstriction channels. Our results showed that colchicine inhibits the migration of most melanoma cells, while for a small cell population, it paradoxically increases their migration and invasiveness. These changes are also accompanied by the formation of stress fibers, compensating for the loss of microtubules. Simultaneous administration of selected agents led to the inhibition of this compensatory effect. Collectively, our results highlighted that colchicine led to actomyosin activation and increased the level of cancer cell invasiveness. We emphasized that a cellular pathway of Rho-ROCK-dependent actomyosin contraction is responsible for the increased invasive potential of melanoma cells in tubulin-targeted therapy.

## 1 Introduction

The formation of metastases at distant locations stands as the primary contributor to mortality in most types of cancers.^1^ During cancerogenesis, cancer cells, in addition to gaining the ability to unrestricted and uncontrolled division, also acquire the ability to form metastases.^2^ During metastasis, the cancer cells must be able to leave the primary tumor, escape through several biological barriers, and then pass through the blood or lymphatic circulation system to new distant locations, where they form secondary tumor foci.^3^ In most cases, the development of the invasive potential of cancer cells is associated with epithelial–mesenchymal transition (EMT).^4^ As a result, cancer cells reduce their adhesion to neighboring cells within the primary tumor and increase their ability to migrate.^5^

Melanoma stands out as one of the most aggressive forms of cancer and the most invasive skin malignancy. Despite representing only 4% of skin cancer cases, melanoma contributes to 80% of deaths in this group.^6^ Melanoma metastasizes to nearby locations in the skin and distinct locations such as the lymph nodes, lungs, liver, and brain.^7,8^ The following stages of melanoma progression have been identified. Stage I (benign and dysplastic nevus) involves small and benign lesions, during which the development of the tumor occurs relatively slowly. Stage II (radial growth phase, RGP) shows the acceleration of tumor progression; however, cell growth is still limited to the dermis layer. Stage III (vertical growth phase, VGP) reveals a significant increase in cell invasiveness by reaching the nearest lymph nodes. Stage IV (metastasis) displays secondary tumor sites at distant locations, demonstrating that cancer cells spread beyond the primary tumor site. VGP melanoma is a poor prognostic factor, significantly reducing the chance of successful patient treatment.^7,9,10^ The strong differentiation of the invasive potential between the individual stages of melanoma development and the extremely high degree of invasiveness observed for the last melanoma stages, combined with the relatively high mortality rate, make melanoma a good research model of cancer invasiveness. The treatment of advanced melanoma covers tumor resection supplemented with radiotherapy, local chemotherapy, or immunotherapy. Both radiotherapy and chemotherapy have been reported to paradoxically promote distant metastasis;^11,12^ however, the mechanism causing this phenomenon remains incomplete. Possible explanations address changes in the tumor microenvironment, the release of cancer stem or stem-like tumor-initiating cells from the primary tumors, and EMT.^13−15^

In the present study, we investigated the effect of colchicine, a drug affecting microtubule integrity, offering a basis for better understanding the reorganization of the cell cytoskeleton and alterations in the invasiveness potential of melanoma cancer cells. We focused on two melanoma cell lines, namely, WM35 (melanoma RGP) and WM266–4 (skin metastasis). The results showed that colchicine significantly increases the invasive potential (defined as cancer cell mobility and active penetration of mechanical barriers) of melanoma cells. By the use of additional molecular inhibitors, we demonstrated that compensatory activation in tubulin-targeted therapy led to the activation of the actomyosin cytoskeleton. We concluded that the Rho-ROCK (Rho-associated protein kinase) pathway is attributed to the increased invasiveness of melanoma cells.

## 2 Materials and Methods

### 2.1 Cell Lines

WM35 melanoma cells were isolated from the RGP (RRID: CVCL_0580). WM266-4 melanoma cells were isolated from skin metastasis (RRID: CVCL_2765). Cell lines have been developed from female donors—WM35 from the 24 year-old donor and WM266-4 from the 55 year-old patient.^16^ The detailed information about cell lines, including WM35 and WM266-4 cells, can be found in Cellosaurus (https://www.cellosaurus.org). Cell lines were obtained from the Chair of Medical Biochemistry at the Collegium Medicum of Jagiellonian University in Krakow (Poland). They were grown in Roswell Park Memorial Institute Medium 1640 (RPMI-1640, Sigma-Aldrich, Poznań, Poland) supplemented with 10% fetal bovine serum (FBS, ATCC, LGC Standards, USA). The cells were cultured at 37 °C in 5% CO_2_. The cells were passaged twice a week until they reached a confluence of 80%. Cell line authentication was routinely conducted using the FTA Sample Collection Kit for the Human Cell Authentication Service (LGC Standards, USA). Cultured cells were treated using molecular inhibitors listed in Supporting Information 1.1. Colorimetric cell proliferation assay (3-(4,5-dimethylthiazol-2-yl)-5-(3-carboxymethoxyphenyl)-2-(4-sulfophenyl)-2*H*-tetrazolium (MTS)) and lactate dehydrogenase assay (LDH) were used to test the cytotoxicity of applied inhibitors and to select the working concentrations (Supporting Information 1.2).

### 2.2 Cell Morphology

Cells were prepared as described in Supporting Information 1.3. Images were acquired in the phase-contrast mode (UCPlanFLN objective, 20×, NA = 0.7) using an inverted microscope (IX83, Olympus with mercury lamp, and Prime BSI Express Scientific CMOS camera: 01-prime-BSI-EXP). The morphology of cells was manually classified into four categories based on their shape: epithelial (E), mesenchymal (M), hybrid (H), and damaged cells (a minimum of 50 cells per condition was analyzed). The percentage of cells with a given morphology was counted and normalized to the total number of cells (damaged and dividing cells were excluded from the analysis).

### 2.3 Single-Cell Migration Assay

Cells were prepared as described in Supporting Information 1.4. The plate with cells was placed in a thermostated CO_2_ incubator (Olympus), providing optimal conditions for live-cell imaging (37 °C, 5% CO_2_, and >95% humidity). CO_2_ is connected to a gas exchange system (Tokai Hit) and combined with an inverted optical microscope (IX83, Olympus). Images of migrating cells were acquired using the videoscopy mode (UPLANFLN objective, 10×, NA = 0.3) operated by the CellSens software (Olympus). Snapshots were captured every 10 min (4 h-long experiment). The recorded images were analyzed using the *Hiro* software (courtesy of the Department of Cell Biology at the Faculty of Biochemistry, Biophysics, and Biotechnology of the Jagiellonian University, Krakow, Poland). Observed cell trajectories are presented as circular diagrams, in which the starting points of the trajectories are reduced to a common origin of the coordinate system.^17^ The mean migration speed and displacement were calculated for each cell. The analysis was conducted for 50 cells from three independent replicates (150 cells in total were analyzed).

### 2.4 Transmigration of Single Cells

The ability of melanoma cells to penetrate through a mechanical barrier was conducted using porous inserts (polycarbonate membrane with a pore diameter of 8.0 μm, Corning Costar Transwell) according to the protocol described in Supporting Information 1.5.

### 2.5 Cytoskeletal Elements

#### 2.5.1 Laser Confocal Fluorescence Microscopy

Tubulin and actin filaments in melanoma cells were visualized using laser scanning confocal fluorescence microscopy (Zeiss LSM 800 AiryScan, 63×, NA = 1.4 oil immersion, light sources: LSM 800 Laser Module URGB, diode lasers 405 nm (5 mW), 488 nm (10 mW), and 561 nm (10 mW)). The setup is equipped with three internal GaAsP photomultiplier (PMT) detectors in reflection mode with tunable emission bands in the 400–800 nm range. The melanoma cells were seeded on the glass bottom of the 24-well plates (SensoPlate, Biokom, Janki, Poland). After 24 h of incubation with the molecular inhibitor, the cells were fixed with a 3.7% paraformaldehyde solution in phosphate-buffered saline (PBS) for 15 min. The cell membrane was permeabilized for 5 min using 0.2% Triton X-100 (Sigma-Aldrich, Poznań, Poland). Microtubules were labeled with antitubulin antibodies 1:200 in PBS (monoclonal anti-α-tubulin antibody; Sigma-Aldrich, Poznań, Poland) for 24 h and stained with antimouse antibodies conjugated with Alexa Fluor 555 (goat anti-mouse IgG (H + L) cross-adsorbed secondary antibody, Invitrogen). The actin filaments were stained with phalloidin conjugated with Alexa Fluor 488 (1:5000 in PBS, Invitrogen) for 15 min. Samples were extensively washed with PBS after each step of labeling. In addition to the cytoskeleton, the cell nuclei were stained with Hoechst 34580 (Sigma-Aldrich, Poznań, Poland).

#### 2.5.2 Quantification of Actin Filaments

Images were acquired using a 100× objective (100× objective, NA = 1.3) with an inverted microscope (IX83), Olympus with a mercury lamp, and a Prime BSI Express Scientific CMOS camera (01-prime-BSI-EXP). The level of actin polymerization was quantified using FilamentSensor2.0 free software,^18^ according to the method described before.^84^ Twenty-two images were collected with a similar acquisition time of 30 ms. Images of representative cells were extracted using *Fiji* software from acquired images.^19^ The number and length of detected filaments per cell were calculated. The minimal length of the fiber was set to 75 px (∼5 μm) to reduce the falsely detected fibers. Fibers detected at the image edges were excluded from the analysis. No additional image filtering was applied to the software.

### 2.6 Atomic Force Microscopy Measurements and Apparent Young’s Modulus Determination

#### 2.6.1 Atomic Force Microscopy Measurements

Cells were seeded on the bottom surface of plastic Petri dishes (TPP, Genos, Łódź, Poland) containing 2 mL of RPMI 1640 medium supplemented with 10% FBS for 48 h before measurements. The cells were cultured in the CO_2_ incubator at 37 °C, 5% CO_2_, and 95% humidity. After 24 h, the culture medium was exchanged with a fresh RPMI 1640 medium containing 1% FBS, which served as the control. To assess the influence of specific cytoskeletal inhibitors on cell deformability, individual inhibitors or their mixtures were added for 24 h at the final concentrations (Supporting Information Table 1). Before AFM measurements, the culture medium (containing inhibitors) was replaced with a fresh one. To maintain a stable pH of 7.4 during the measurements, HEPES (4-(2-hydroxyethyl)-1-piperazineethanesulfonic acid, Sigma-Aldrich, Poznań, Poland) was added to achieve a final concentration of 25 mN. The measurements were conducted using AFM (CellHesion 200 head, Bruker—JPK Instruments) at 37 °C maintained by a PetriDish heater (Bruker-JPK Instruments). V-shaped cantilevers with a nominal spring constant of 0.03 N/m were used (MLCT-BIO–DC-D, Bruker). Before measurements, the cantilever sensitivity was calibrated using a Petri dish surface without cells. The spring constant of each cantilever used was calibrated using the thermal tune.^20^ For each sample (cell type and experimental conditions), 10–20 cells were measured using the force–volume mode in the nuclear area (5 × 5 μm^2^). For each cell, 25 force–distance curves were acquired (loading force: 2 nN, loading rate: 8 μm/s, and *z* range: 5–6 μm). Each sample was measured within 60–90 min. Each experiment was conducted in triplicate.

#### 2.6.2 Elastic Modulus Determination

Apparent Young’s modulus was calculated using JPK Processing Software (JPK Systems/Bruker). First, the calibration curve was subtracted from each curve acquired on living cells. As a result, a relation between the load force and indentation depth was obtained, which was analyzed using the Hertz–Sneddon contact mechanics, assuming that a cone can approximate the shape of the probing tip.^21^ For such geometry, the relation between load force *F* and indentation depth δ is as follows:
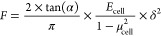
where α is the half angle of the probing tip, *E*_cell_ is the apparent Young’s modulus of the cell, and μ is Poisson’s ratio (assumed to be equal to 0.5 for incompressible materials). For each measured cell, the average value was calculated. Then, the final apparent Young’s modulus was expressed as a mean and standard deviation from all measured cells within a specific group.

### 2.7 Sample Preparation for Microfluidic Measurements

Relative differences in mechanical properties of the cells subjected to drug exposure were determined as the transit time of cells through constriction channels (10 × 12 μm^2^ cross-section, 300 μm long) within a microfluidic bifurcated network with bypass channels. The device was designed based on Rosenbluth and co-workers,^22^ adapted to support 8 parallel, 300 μm-long constriction domains, and with 155 μm-wide bypass channels. These microfluidic devices were fabricated by a soft lithography approach implemented in a multi-user cleanroom (NTNU NanoLab) with process steps as described previously^23^ and adapted to this experiment (Supporting Information 1.6).

### 2.8 Statistical Analysis

The analysis of the statistical significance of differences was carried out using OriginPro 2022 software. First, all obtained data sets were verified for normality of the distribution using the Shapiro–Wilk test. For Gaussian distributions, the statistical analysis was conducted using the ANOVA method with post hoc multiple comparisons Dunnett’s test. For non-Gaussian distributions, we used the Kruskal–Wallis test with post hoc multiple comparisons Dunn’s test. To compare only two sets of data, Student’s *t*-test (for parametric values) or the Mann–Whitney test (for nonparametric values) was applied (notation: *p < 0.05; **p < 0.01; and ***p < 0.001).

## 3 Results

### 3.1 RGP and Skin Metastasis Melanoma and Skin Cells Exhibit Different Morphological Phenotypes Manifested in Distinct Mechanical and Migratory Properties

**Figure 1:**
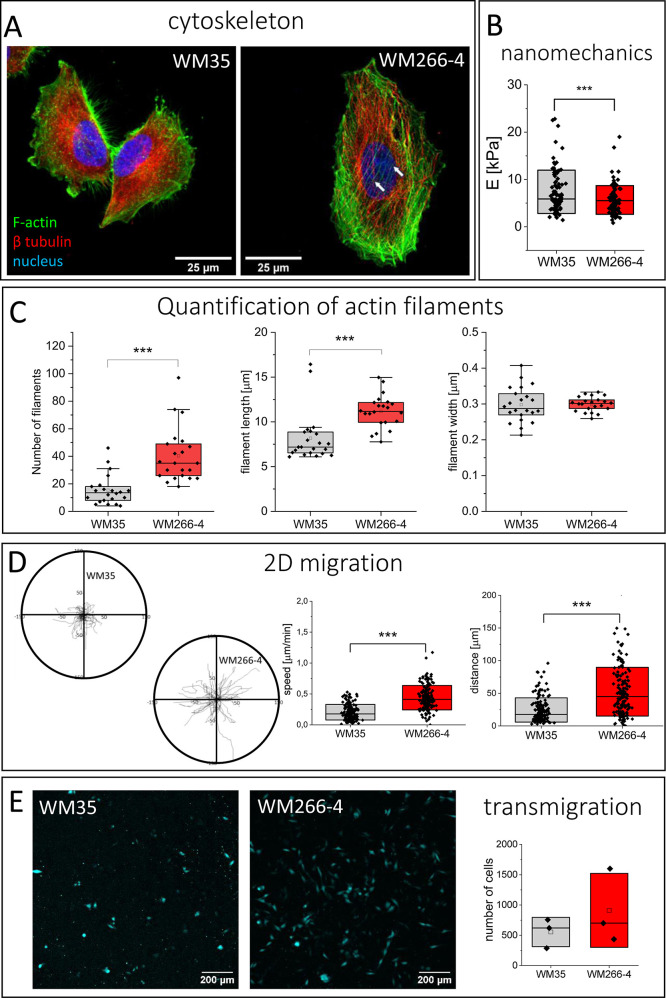
Melanoma cells display a heterogeneous population of individual cells with different morphologies and mechanics. (A) Organization of actin filaments and microtubules (a white arrow indicates stress fiber) visualized by fluorescence microscopy. (B) Apparent Young’s modulus obtained for WM35 (*n* = 90 cells) and WM266-4 (*n* = 97 cells) cells measured by AFM. (C) Quantification of actin filaments based on fluorescence microscopy using FilamentSensor software. Data on number, length, and width of detected actin filaments was presented for WM35 (*n* = 22 cells) and WM266-4 (*n* = 22 cells) cells. (D) Heterogeneity of melanoma cell lines is visible in the 2D migration. Migratory trajectories of WM35 and WM266-4 cells quantified by mean values of migration speed and distance were presented. (E) Transmigration efficiency calculated by tracing fluorescently labeled cells passing through pores of 8 μm in diameter.

To assess the heterogeneity in the studied populations of WM35 (RGP) and WM266-4 (skin metastasis) melanoma cells, we first started with the identification of morphological differences by optical microscopy. The distinct morphology of melanoma cells manifested in the specific organization of actin filaments and microtubules as visualized with confocal fluorescence microscopy (Figure 1A). Thick actin bundles (i.e., stress fibers) were identified only in WM266-4 melanoma cells (Figure 1A and Supporting Information Figure 1). WM-266-4 cells have more actin filaments than WM35 cells; the filaments were longer, but the widths of the filaments were similar in both cell lines (Figure 1A,C). The polarization of melanoma cells (defined as a ratio of the long and short axis of the cell) was observed for WM35 and WM266-4 cells (Supporting Information Figure 2C). Moreover, the two cell lines can be distinguished based on epithelial–mesenchymal-like (EMT-like) transition (Supporting Information Figure 2). In particular, 70% of cells derived from skin metastasis were classified as mesenchymal-like, in contrast to 30% for cells derived from RPG (Supporting Information Figure 2B).

The difference in the organization of actin filaments can be attributed to changes in cell mechanical properties and quantified by apparent Young’s modulus (a measure of cell deformability), as already revealed from the AFM measurements of single cells;^24,25^ therefore, we conducted nanoindentation measurements of melanoma cells (Figure 1B). The results indicated a trend toward increased deformability (lower values of apparent Young’s modulus) of WM266-4 cells than WM35 cells (*p* = 0,106). Apparent Young’s modulus values were 7.3 ± 0.5 kPa (*n* = 90 cells) and 5.5 ± 0.3 kPa (*n* = 97 cells), respectively. Moreover, the proliferative efficacy, evaluated by the rate of cell division, of WM266-4 was higher than that of WM35 cells (Supporting Information Figure 3).

Alongside morphological features, cells’ migratory properties were evaluated in terms of cell speed and displacement (during 4 h) (Figure 1C); for WM35 cells, the mean values were 0.21 ± 0.13 μm/min (*n* = 150 cells) and 24.5 ± 18.6 μm (*n* = 150 cells), while for WM266-4 cells, they were 0.44 ± 0.20 μm/min (*n* = 150 cells) and 52.4 ± 37.4 μm (*n* = 150 cells). Despite large statistical significance, there is large variability in migration speed and distance (Figure 1D), indicating a fraction of slow and fast migrating cells in both melanoma cell types.

The crucial aspect of the invasive potential of cancer cells is their ability to penetrate various barriers.^26,27^ To mimic cancer cell transmigration through the mechanical barrier, we used a porous polycarbonate membrane as a model. The analysis of the invasive potential of melanoma cells showed no statistically significant differences between the number of cells passing the mechanical barrier for both cell lines (Figure 1E). To ensure that cell transmigration is not influenced by differences in the size of the nuclei, we compared the size of the nuclei between WM35 and WM266-4 cells by cellular staining. These results showed no statistically significant differences between these cell lines (WM35 198 ± 74 μm^2^ vs 190 ± 104 μm^2^ in WM266-4 cells), indicating that these differences should not influence the differences in cell transmigration. Overall, we can conclude that both studied melanoma cells differ in terms of morphology, accompanied by changes in their mechanical and migratory properties.

### 3.2 Impact of Drugs on the Ability of Cells to Penetrate Mechanical Barriers

**Figure 2:**
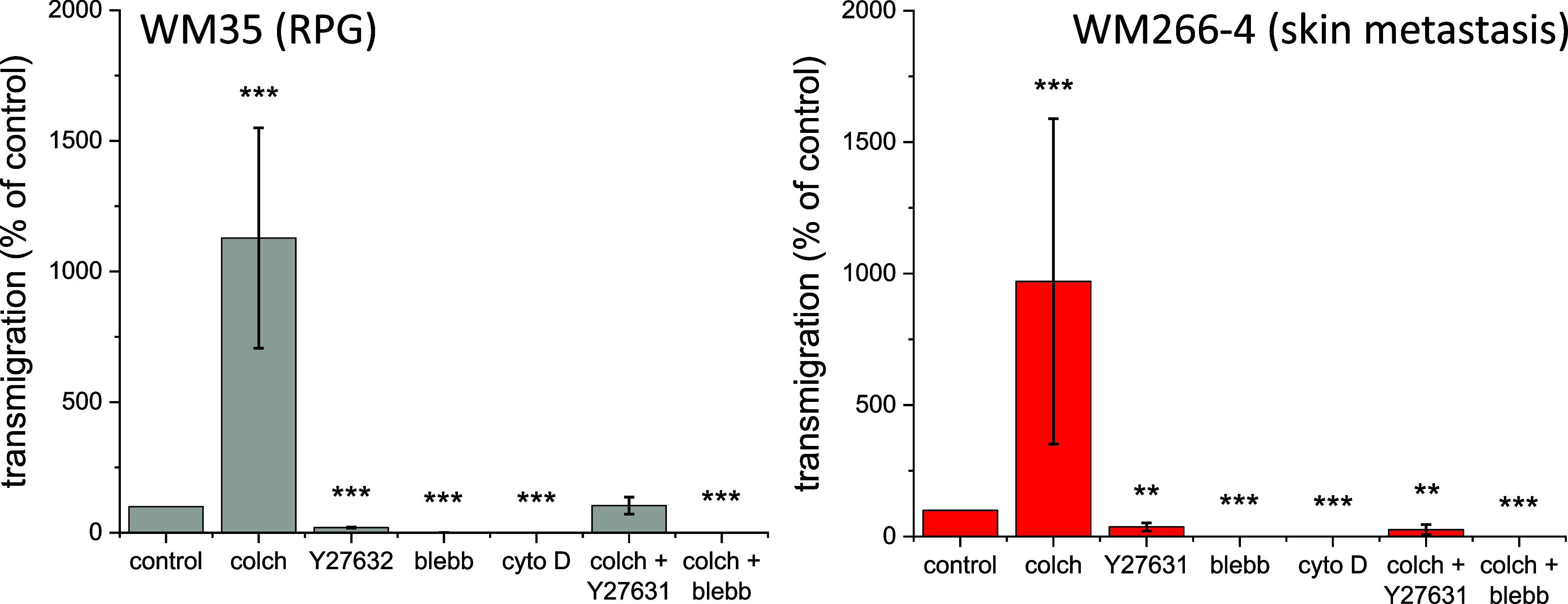
Transmigration through a porous polycarbonate membrane for WM35 (RGP) and WM266-4 (skin metastasis) melanoma cells. The data are expressed as a relative ratio [%] of fluorescently labeled cells that passed through the membrane after 24 h of culture. (Representative images are presented in the Supporting Information.)

The most deadly feature of cancer is the ability of cells to leave the primary site and form metastases. Thus, in the next step, we quantified the ability of cells to pass through the mechanical barrier (8 μm pores) in the presence of drugs. First, we assessed the dose using MTS and LDH tests (Supporting Information Figures 4–6). Then, in the presence of chosen concentrations of the inhibitors, we conducted transmigration experiments (Figure 2 and Supporting Information Figure 7).

The results showed a nearly 10-fold increase in the number of transmigrating cells treated with colchicine, regardless of the melanoma type, compared to untreated control cells. Furthermore, blebbistatin and cytochalasin D arrested the ability of cells to pass through porous membranes. Y27632 significantly reduced the transmigration rate. The effect was inhibitor and cell-type dependent for the mixture of a selected molecular inhibitor with colchicine. Colchicine + blebbistatin followed the results of blebbistatin only, while colchicine + Y27632 revealed cell-specific transmigration, which was unaltered for WM35 cells and decreased for WM266-4 cells.

### 3.3 Response of the Melanoma Cytoskeleton to Drug Treatment

#### 3.3.1 Changes in the Cytoskeleton—Compensation of Disrupted Tubulin by Actin Polymerization

**Figure 3:**
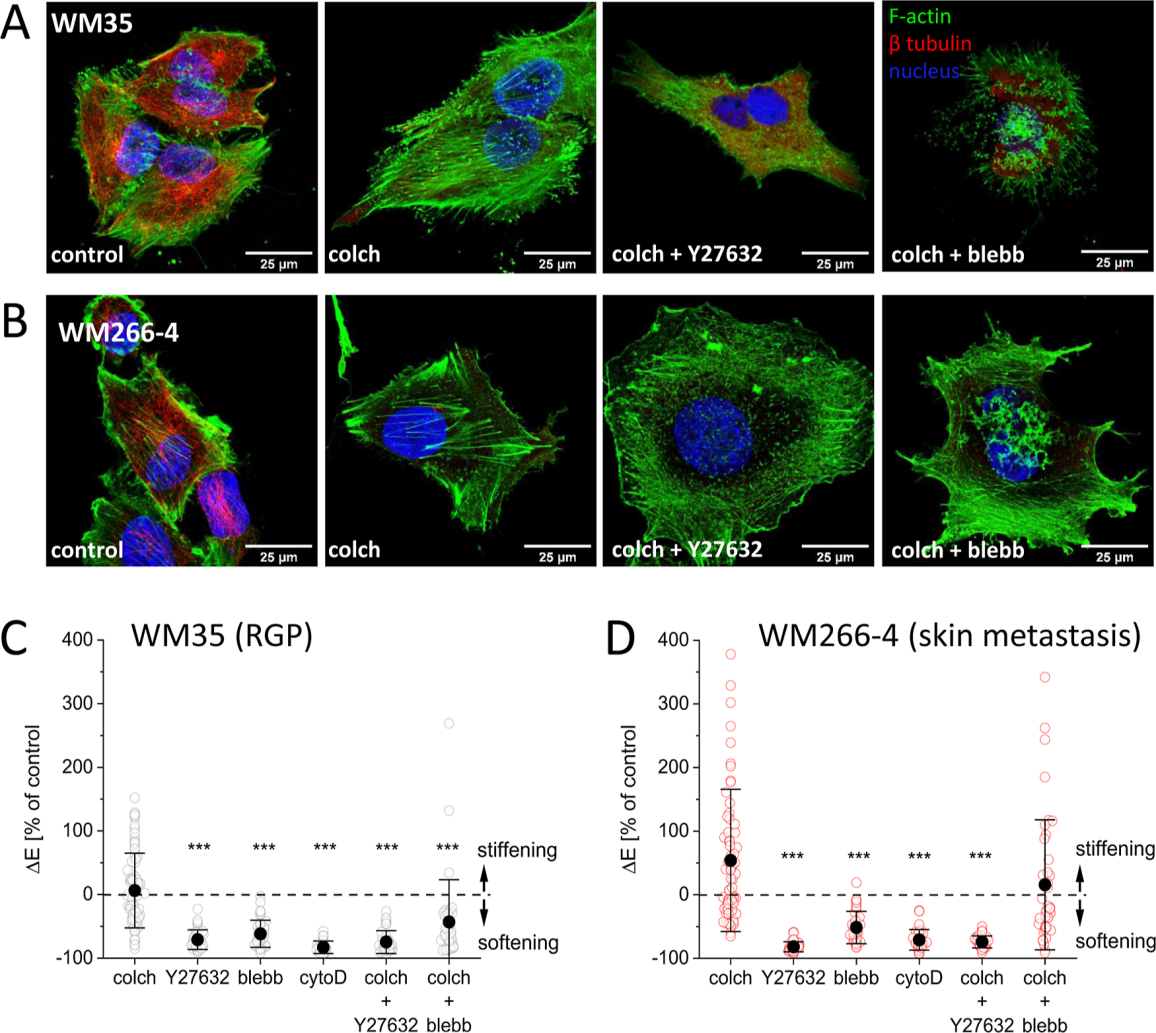
Response of the melanoma cytoskeleton to the drug treatment. (A) WM35 and (B) WM266-4 melanoma cells after 24 h of treatment with colchicine, colchicine + Y27632, and colchicine + blebbistatin. Actin filaments (F-actin, Alexa Fluor 488), microtubules (β tubulin, Alexa Fluor 555), and cell nuclei (Hoechst 34580). (C) Changes in mechanical properties of drug-treated (24 h) WM35 (RGP) and (D) WM266-4 (skin metastasis) melanoma cells measured using AFM. Apparent Young’s modulus was calculated for a load force of 2 nN (resulting in an indentation depth of 500–1000 nm).

To visualize the cell cytoskeleton, actin filaments and microtubules were fluorescently stained before and after the treatment with colchicine, blebbistatin, Y27632, cytochalasin D, combined treatment with colchicine + Y27632, and colchicine + blebbistatin (Figure 3 and Supporting Information Figures 8–11).

Colchicine induces microtubule depolymerization, and this effect has been documented in melanoma cells.^28^ Here, we showed that the microtubular network was disorganized, and the fluorescence signal diffused (Figure 3 and Supporting Information Figures 8 and 9). The microtubule depolymerization was accompanied by the formation of thick actin bundles in both melanoma types. The effect was more pronounced for WM35 cells (which, unlike WM266-4 cells, do not have stress fibers under control conditions). The cell treatment with Y27632 or blebbistatin did not affect the microtubular network in both cell types (Supporting Information Figures 10 and 11). The exception is cytochalasin D, which induced strong depolymerization of the actin cytoskeleton, leading to cell shrinking. As a result, the microtubular network changed its organization, but existing microtubules were not depolymerized. Colchicine + blebbistatin and colchicine + Y27632 led to the disruption of microtubules, similar to the treatment with colchicine alone. In the case of WM35 cells, the formation of actin stress fibers was also blocked. WM266-4 cells (which had stress fibers in the control, untreated group), incubated in drug cocktails, presented rudimentary stress fibers that were present on the cell peripheries.

#### 3.3.2 Changes in Melanoma Cell Mechanics Induced by Drug Treatments

Changes in the organization of the cytoskeleton are often attributed to changes in the mechanical properties of the cells. We conducted experiments to quantify the changes in cell elasticity in response to selected drugs. First, we compared apparent Young’s modulus for mesenchymal, hybrid, or epithelial cells (Supporting Information Figure 12). No significant differences were observed in the deformability of these groups. The cells of both lines were incubated in the presence of a drug or a drug cocktail for 24 h (Figure 3C,D). Negative values in relation to the control indicate cell softening (i.e., larger deformability), while positive values denote cell stiffening. Except for colchicine, all molecular inhibitors applied induced cell softening, regardless of the cell type. Colchicine + Y27632 manifested in cell softening in both cell types, indicating that the ROCK inhibitor attenuated the formation of stiff actin fibers. Colchicine + blebbistatin resulted in the elasticity values of untreated melanoma cells. However, for WM35, slight softening could be detected, despite large modulus variability. For skin melanoma (WM266-4) cells, there was no significant change after colchicine + blebbistatin as compared to the control. This study showed that colchicine did not cause statistically significant changes in elasticity in WM35 and WM266-4 cells. It indicates that newly formed thick actin bundles took over the biomechanical function of the cells.

#### 3.3.3 Transition of Melanoma Cells through Constriction Channels

**Figure 4:**
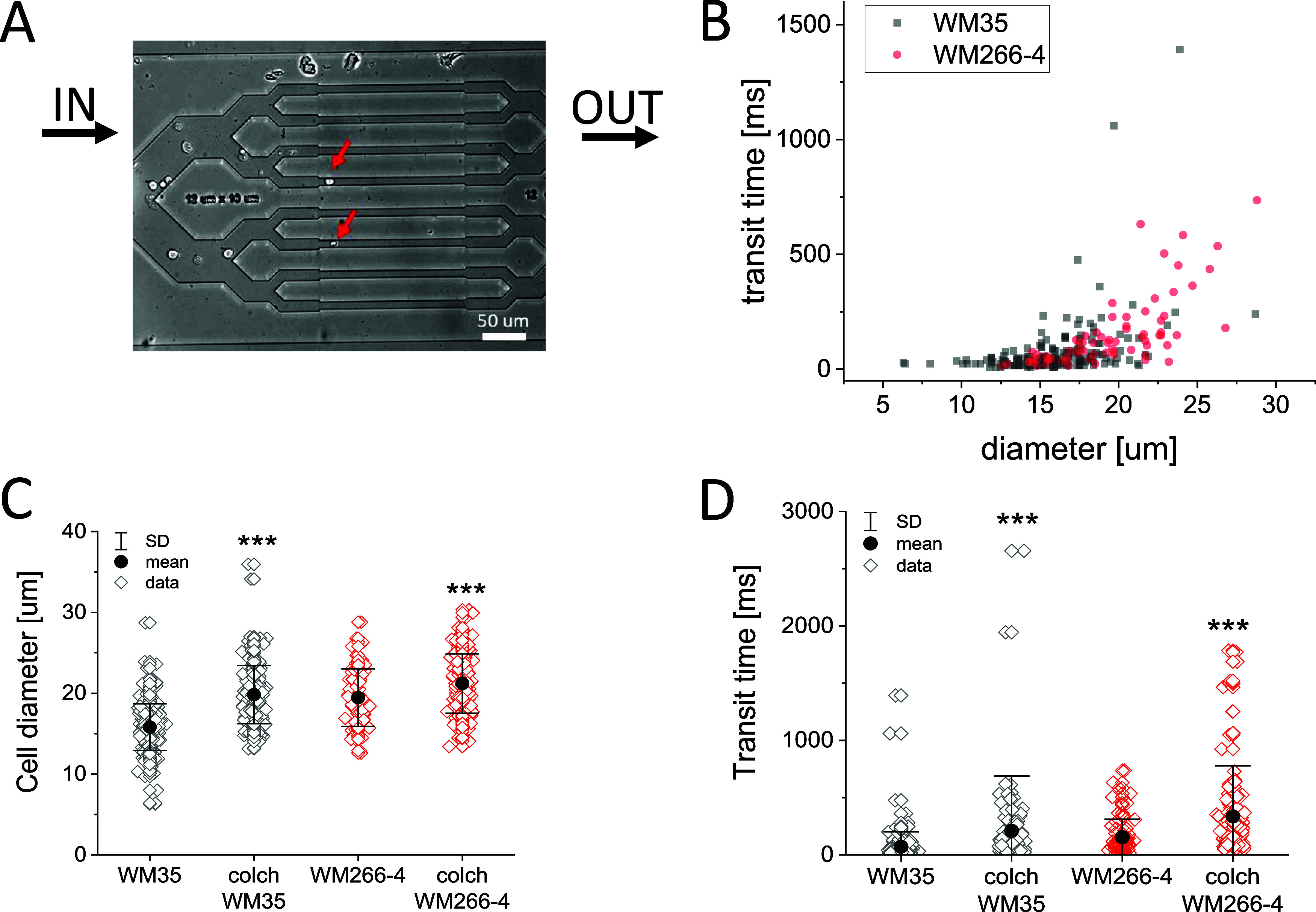
Transition of WM35 (RGP) and WM266-4 (skin metastasis) melanoma cells through constriction channels. The forced transition of the cells was mediated by an overall volumetric flow *Q* = 150 μL/min over the bifurcated microfluidic device. (A) Individual frame showing melanoma cells passing through the channel (red arrows indicate cells passing through the channel), which was quantified by transition time. (B) Dependence of the transition time on cell diameter. (C) Distributions of cell diameter and (D) transition time for control and colchicine-treated melanoma cells. Number of cells analyzed; WM35 control: *n* = 208 cells; WM35 + colchicine: *n* = 159 cells; WM266-4 control: *n* = 71 cells; and WM266-4 + colchicine: *n* = 147 cells.

Parallel to the determination of apparent Young’s modulus values by AFM, the transition time of the cells through well-defined constriction channels with cross-sections less than the size of the melanoma cells (rectangular cross-section 10 μm × 12 μm and length 300 μm) was measured (Figure 4). The cell suspension was subjected to a volumetric flow in a bifurcated microfluidic network, yielding relative transit times that reflect relative mechanical properties (Figure 4A).

The transition time data revealed the effect of the cell size (Figure 4B). Analyzing individual cells, we noticed that some cells were >2 times larger than the mean cell size (Figure 4B,C). We addressed this observation using fluorescence imaging and identified a fraction of polyploid giant or dividing cells (Supporting Information Figure 13). Cells of both cell lines tend to increase the cell diameter after colchicine treatment (Figure 4C). This, in turn, caused an increased mean transit time (Figure 4D).

### 3.4 Migration of WM35 and WM266-4 Melanoma Cells

**Figure 5:**
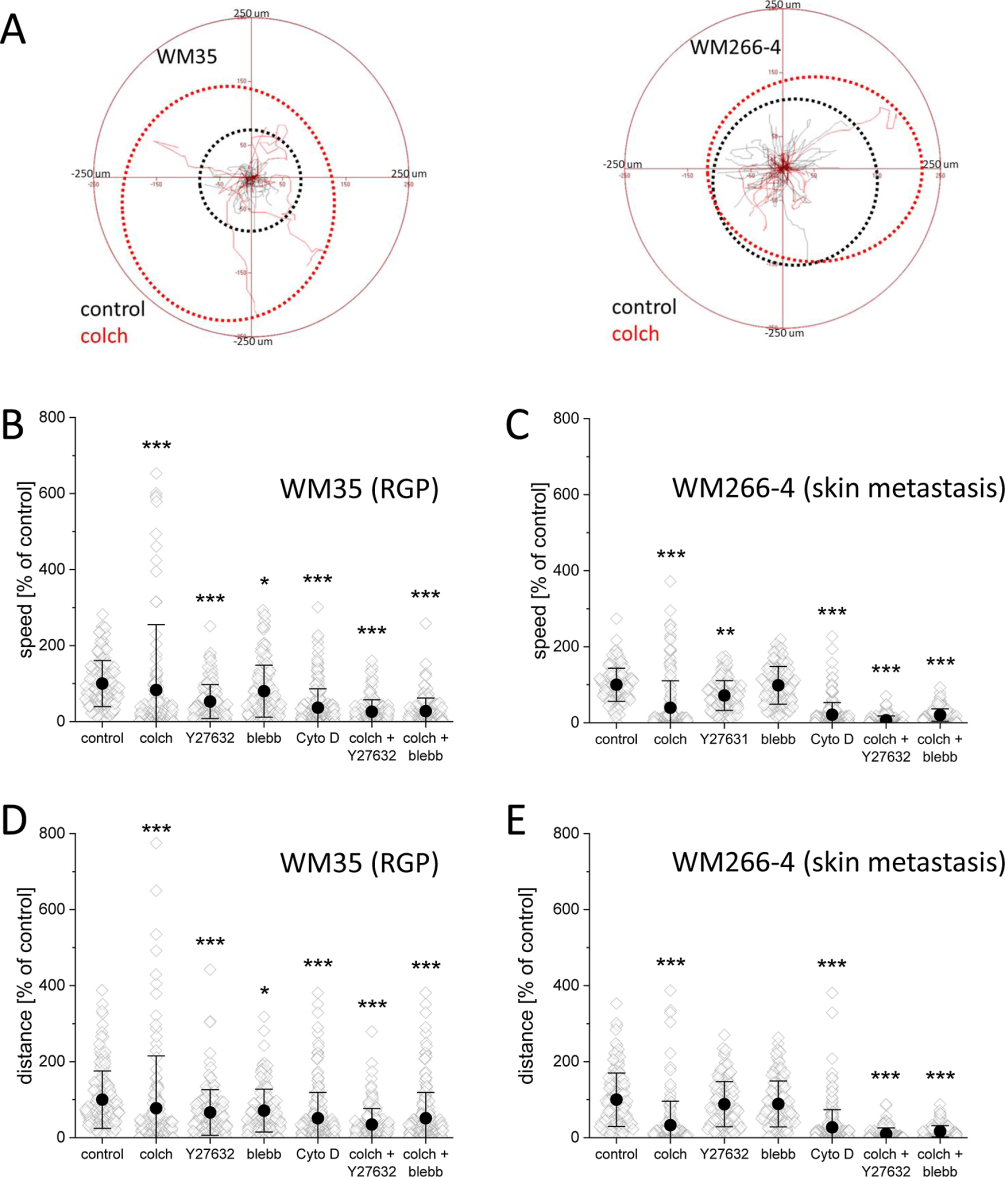
Migration of WM35 (RGP) and WM266-4 (skin metastasis) melanoma cells. (A) Circular plots show how far melanoma cells move before (black dotted perimeter) and after (red dotted perimeter) colchicine treatment. (B,C) Dot plots present the speed of migration and (D,E) total displacement of individual cells.

The characteristic feature of cells responsible for the invasive potential is their ability to actively migrate. Therefore, here we focused on the impact of the drugs on the motility of WM35 and WM266-4 cells using time-lapse microscopy (Figure 5 and Supporting Information Figure 14).

Colchicine led to a strong inhibition of the migration activity in most melanoma cells in both cell lines. Although the migration of cells was significantly reduced, a small subpopulation of cells with activated migration was observed (red dotted perimeter in Figure 5A). Moreover, the level of activation of the melanoma cell line representing RGP was more profound than melanoma from skin metastasis, reaching similar displacement after treatment. Y27632 only partially reduced (47% ± 3.6% for WM35 and 29% ± 3.2% for WM266-4 cells) the migration speed of WM35 and WM266-4 cells, while blebbistatin had no significant effect (Figure 5B–D). Cytochalasin D led to a major (>60%) reduction in the migration activity of cells in both cell lines. Colchicine + Y27632 or colchicine + blebbistatin strongly inhibited the migration activity of melanoma cells (>70% for WM35 and >90% for WM266-4).

## 4 Discussion

In this study, we selected two melanoma cell lines, representing RGP (WM35) and skin metastasis (WM266-4), to investigate the effect of tubulin-targeted colchicine therapy on the bimodal effect on cell invasiveness.

### 4.1 Characteristics of WM35 (RGP) and WM266-4 (Skin Metastasis)

The thorough analysis of several cell properties allowed us to highlight changes in melanoma cells associated with cancer development. We summarize that the selected cell lines represent the different stages of melanoma. WM266-4 cells were characterized by significantly higher proliferative and migratory activity, higher ability to transmigrate through biological barriers, and the presence of prominent actin filaments as compared to WM35 cells. These findings reflect the observations in previous reports, as increased migratory properties are linked with the formation of metastases by cancer cells.^29,30^ In addition, the classification of cell morphology, according to the previous method,^31,32^ showed that WM266-4 cells have a much more mesenchymal character than WM35. This finding shows that, among other changes, an EMT-like transition occurs with the development of melanoma, which leads to malignancy of the tumor. This process occurs not only in melanoma^33^ but also in other types of cancers and is characterized by an increase in their invasive potential.^34−36^

### 4.2 Colchicine-Targeted Therapy May Lead to Increased Invasiveness of Cancer Cells

Colchicine is a compound with a strong inhibitory effect on the polymerization of microtubules. Its mechanism of action involves microtubule-based inflammatory cell chemotaxis, altered production of eicosanoids and cytokines, and phagocytosis, but it still remains under study.^37^ Colchicine is medically approved to treat gout^38,39^ and familial Mediterranean fever.^40^ It was reported to be beneficial in cardiovascular diseases,^41^ liver fibrosis, and inflammation.^42^ Additionally, due to its tubulin-targeted mechanism of action, colchicine has been postulated for use in the treatment of cancer diseases or in reducing cancer invasiveness.^43−52^ In this report, we presented the side effect of colchicine on the biological activity of WM35 and WM266-4 cancer cell lines, showing a bimodal effect on the migratory properties. Our experiments show that colchicine led to a significant decrease in the proliferative activity of melanoma cells and reduced migratory activity of most cells; however, an emphasis should be drawn to the behavior of a fraction of the cell population characterized by increased invasiveness after treatment. We address the presented observation to previous reports in which radiotherapy and chemotherapy have been reported to paradoxically promote distant metastasis.^11,12,15^ We emphasize that in our experiments, the activation effect of cell migration reaches the same level in both cell lines, indicating that initially, less active cells from RGP became as active as metastatic cells after colchicine treatment. We highlight here that the diagnosis of cancer should precede therapy based on colchicine. This report aims to characterize and understand this phenomenon on a cellular level, focusing on the cytoskeleton.

### 4.3 Tubulin Disruption Causes Crosstalk to the Actomyosin Network

We hypothesize that the observed effect of increased invasiveness of colchicine-treated cells involves cytoskeletal crosstalk between microtubules and the actomyosin network and that the compensation by actomyosin is mechanistic for increased cell mobility. To test this hypothesis, in addition to colchicine, we introduced two molecular inhibitors targeting the actomyosin cytoskeleton, namely, Y27632 and blebbistatin, which are ROCK inhibitors and myosin II ATPase inhibitors, respectively. GTPases, such as Rho, Rac, and cdc42, regulate multiple cytoskeletal processes,^53,54^ including cell migration.^55^ In particular, activating the Rho-ROCK pathway results in phosphorylation of the myosin regulatory light chain (RLC), which drives myosin contractility. As a result, ROCK induces myosin contraction and promotes cell motility. Additionally, the Rho-ROCK pathway participates in the direct regulation of actin-binding proteins, such as profilin, cofilin, and gelsolin, and may result in the rearrangement of short actin mesh into thick actin fibers.^54,56−59^ Inhibition of ROCK was reported to attenuate cancer cell motility.^60,61^ The second molecular inhibitor, blebbistatin, bypasses the regulation via RLC and acts directly by binding to the motor domain of a myosin-heavy chain. Therefore, it was used here to uncouple the regulation of actomyosin by GTPases and to investigate only the myosin component of the actomyosin network.^54^

Although the cellular microtubules are disrupted due to colchicine treatment, the compensation in the increased number of actin stress fibers was significant. This effect may also be caused by GEF-H1 (guanine nucleotide exchange factor H1) release. This factor was shown to be associated with microtubules and regulated by the polymerization state of microtubule networks.^62^ As a result of tubulin disruption, it can be released and induce activation of Rac1 and RhoA GTPases.^63,64^ GEF-H1 release from microtubules was indicated to promote activation of the RhoA-ROCK-myosin pathway, which increases actin polymerization and acto-myosin contraction.^65^ In the case of various cancer types, changes occurring within the cytoskeleton of cells lead to a change in their invasiveness.^66−68^ More studies are needed to evaluate the role of GEF-H1 related to the observed effect of colchicine on melanoma cells. Drugs affecting the actin cytoskeleton^69,70^ or microtubules^71,72^ usually reduce the invasive potential of cancer cells. On the other hand, it is also known that affecting one type of filament can lead to changes in the remaining filament types;^73−75^ such an effect is also known in the case of colchicine, where the formation of stress fibers compensates the disruption of microtubule formation.^76^ However, so far, it has never been demonstrated that colchicine can increase the invasiveness of cancer cells. Our study is not only the first to show that this drug can have such an effect but also confirms how important it is to take into account the crosstalk of microtubules and actomyosin networks in studies on the invasiveness of cancer.

### 4.4 Targeting Actomyosin Network Relaxation Attenuates the Side Effects of Colchicine

Here, the inhibition of ROCK resulted in cell relaxation in both melanoma cell lines, which was sufficient to reverse the effect of colchicine. Alone, Y27632 did not affect the proliferative or migratory activity of the melanoma cells. Moreover, it did not cause any significant changes in the morphology of the cells. However, both Y27632 and colch + Y27632 significantly limited the ability of melanoma cells to penetrate mechanical barriers. Our data from fluorescence microscopy highlight the reduction in the abundance of actin fibers after Y27632 and colchicine + Y27632. The results confirm the complex effect of ROCK on the actomyosin network and highlight the activation of GTPases to compensate for disrupted microtubules. In order to understand whether the reduced migratory properties after Y27632 resulted from changes in actin or myosin, we also performed additional experiments with blebbistatin. Blebbistatin significantly reduced the 2D invasiveness of the WM35 cell line but not that of the WM266-4 cell line. However, active 3D migration was arrested for both cell lines in response to blebbistatin. Most importantly, the side effect of increased motility after colchicine vanished at a similar rate for colchicine + blebbistatin and colchicine + Y27632.

Finally, the importance of the actin cytoskeleton in the invasiveness of melanoma cells was tested with the use of cytochalasin D. This compound is an inhibitor of the interactions of cofilin and actin, thus blocking the polymerization and reorganization of actin filaments.^77,78^ Our study showed that cytochalasin D has cytostatic and cytotoxic effects (with longer exposure), leading to the shrinkage of melanoma cells and blocking their ability to transmigrate through mechanical barriers. Nevertheless, it could be observed that there was no reorganization of microtubules under the influence of cytochalasin D; moreover, the cell shape was adapted to the existing tubulin network. A recent report showed that pretreatment with cytochalasin prevented myosin contraction triggered by myosin light-chain phosphatase (MLCP) inhibitors, i.e., calyculin A.^59^ It was explained that actin fibers facilitate myosin contractility. Disruption of actin fibers prevents myosin from exerting the significant force necessary for cell contraction. These findings potentially explain the inhibited melanoma cell transmigration observed here, as efficient myosin contraction relies on intact actin fibers.

### 4.5 Link between Cell Stiffness and Cell Invasiveness

The differences in cell shape (more mesenchymal character) are reflected in the apparent Young’s modulus of cells as determined by AFM and corroborated by transit time in constriction channels and transmigration rate. It is well established that the actin cytoskeleton regulates cell mechanics.^79−81^ The drastic decrease in the values of apparent Young’s modulus after cytochalasin D and arrested transmigration confirm these findings. Interestingly, we showed that ROCK inhibition reduces the apparent Young’s modulus value and motility function of cells at a similar level to cytochalasin D but with a mild effect on cell morphology. This observation indicates an important contribution of myosin to the deformability of cells. Indeed, we observed a reduction of apparent Young’s modulus after treatment with blebbistatin, similar to others.^82,83^ The drastic reduction of apparent Young’s modulus after ROCK inhibition may be due to changes in both components of the actomyosin network. Still, our observations (the effect of Y27632 and cytochalasin D) indicate the dominant effect of actin, rather than myosin, on apparent Young’s modulus of cells in both lines, but the myosin component seems to be dominant in the regulation of transmigration.

## 5 Conclusions

Thoroughly examining the observed outcomes through diverse experimental assays, specific molecular inhibitors, and two distinct cell lines representing various cancer stages enabled us to furnish in vitro insights into the impact of the cytoskeleton on melanoma invasiveness during treatments involving the tubulin-targeted drug colchicine. We explored the interplay between tubulin and the actomyosin network, proposing that supplementary therapy may be prudent to mitigate the side effects of activated migration, as observed in a subset of cells. Our findings showed that the relaxation of either actin or myosin efficiently vanishes the side effects of colchicine. Finally, we demonstrated that the drug-induced reduction of apparent Young’s modulus (relaxation of cells) correlated with the attenuated migration rate in melanoma cells.

## Data Availability

The data presented in this study are available on request from the corresponding authors.

## Supporting Information Available

Additional experimental details, materials, methods, fluorescence images of the WM35 cell line, different morphology of WM35, cells’ division rate, cytostatic and cytotoxic effects, morphology of WM35 and WM266-4 cells, transmigration through Transwells® of WM35 and WM266-4 cells, organization of actin filaments and microtubules of MW35 cells and MW266-4 cells, AFM data on Young’s modulus, presence of polyploid giant cancer cells, and distribution of movement speed of WM35 and WM266-4 cells (PDF)
